# Supplementary material for: A Bilingual Dataset for Testing Web Probing in the US and India: The Example of Measures of Environmental Concern
Source: J Open Psychol Data. 2024 Dec 6;12:13. doi: 10.5334/jopd.113 (PMC12270270; doi:10.5334/jopd.113)
Supplement: Appendix A. — Exact wording of questions and text elements in the questionnaire. [file jopd-12-113-s1.pdf]

Appendix A: Exact wording of questions and text elements in the questionnaire

Question	Exact Text	Scale	Type of question/ text	Source	Construct	response categories
	Thank you for taking part in this survey on environmental change.<br>In this survey, we will ask you about your opinions regarding the environment, including climate change.<br>This survey is part of a scientific research project that is being carried out by the University of Utrecht in the Netherlands. We will ask people from the US and India. The survey will take 15-20 minutes to answer.<br>All answers are completely anonymous, and cannot be traced back to you. All data will be processed in accordance with the European General Data		Welcome screen	-	-	-

	Protection Regulation (GDPR)					
	Please consider the following: We are interested in your personal opinion. There are no wrong or right answers. Please select the option that comes closest to your personal opinion.<br>The survey is unusual in the way that, for some questions, we ask you to provide reasoning for your answer or to describe what you had in mind when answering. For other questions we ask you how you interpret certain terms. Please take some time to answer these open questions. Your answers will help us to better understand the data which we collect.		Instructions	-	-	-
	We would like to start with some questions about the environment.		Introduction first part	-	-	-
Q1	Which of these statements comes closest to your own point of view?	3	Closed-ended (open if “other” option selected)	WVS5	-	-Protecting the environment<br>-Economic growth should be priority

						-Other, please specify
Q2	To which extent do you agree or disagree with the following statement?<br>I would give part of my income if I were certain that the money would be used to prevent environmental pollution	4-point Likert	Closed-ended	WVS5	WTP	-Strongly agree<br>-Agree<br>-Disagree<br>-Strongly Disagree<br>-Don't know
Q3	Which type(s) of environmental pollution were you thinking of when you answered the previous question?		Open-ended SP	-	WTP	-
Q4	What percentage of your income would you be willing to give if you were certain that the money would be used to prevent environmental pollution?	1-100	Open-ended (number 1-100)	-	WTP	-
Q5	And to which extent do you agree or disagree with the following statement?<br>I would agree to an increase in taxes if the extra money were used to prevent environmental pollution.	4-point Likert	Closed-ended	WVS5	WTP	-Strongly agree<br>-Agree<br>-Disagree<br>-Strongly Disagree<br>-Don't know
Q6	Why did you select [response question 5] when answering		Open-ended CSP	-	WTP	-

	the previous question?					
Q7	What percentage of tax increase would you accept if the extra money were used to prevent environmental pollution?		Open-ended	-	WTP	-
Q8	And to which extent do you agree or disagree with this statement?<br>The government should reduce environmental pollution, but it should not cost me any money.	4-point Likert	Closed-ended	WVS5	WTP	-Strongly agree<br>-Agree<br>-Disagree<br>-Strongly Disagree<br>-Don't know
Q9	Why did you select [response question 8] when answering the previous question?		Open-ended CSP	-	WTP	-
	Now we will ask you a few questions about environmental problems facing many communities. Please select how serious you consider each problem to be in your own community.		Introduction local communities	WVS5	-	-
Q10	How serious do you consider the problem of poor water quality to be in your own community?	4-point Likert	Closed-ended	WVS5	Local environmental problems	-Very serious<br>-Somewhat serious<br>-Not very serious<br>-Not serious at all<br>-Don’t know
Q11	What do you consider to be		Open-ended	-	Local	-

	“poor water quality”?		CP		environmental problems	
Q12	And how serious do you consider the problem of poor air quality to be in your own community?	4-point Likert	Closed-ended	WVS5	Local environmental problems	-Very serious<br>-Somewhat serious<br>-Not very serious<br>-Not serious at all<br>-Don’t know
Q13	What do you consider to be “poor air quality”?		Open-ended CP	-	Local environmental problems	-
Q14	And how serious do you consider the problem of poor sewage and sanitation to be in your own community?	4-point Likert	Closed-ended	WVS5	Local environmental problems	-Very serious<br>-Somewhat serious<br>-Not very serious<br>-Not serious at all<br>-Don’t know
Q15	What do you consider to be “poor sewage and sanitation”?	4-point Likert	Open-ended CP	-	Local environmental problems	-
	Now let’s consider environmental problems in the world as a whole		Introduction global problems	WVS5	-	-
Q16	How serious do you consider the problem of global warming or the greenhouse effect to be for the world as a whole?	4-point Likert	Closed-ended	WVS5	Global environmental problems	-Very serious<br>-Somewhat serious<br>-Not very serious<br>-Not serious at all<br>-Don’t know
Q17	Which problems relating to global warming or the greenhouse effect did you		Open-ended SP	-	Global environmental problems	-

	thinking of when answering the previous question?					
Q18	And how serious do you consider the problem of loss of plant or animal species or biodiversity to be for the world as a whole?	4-point Likert	Closed-ended	WVS5	Global environmental problems	-Very serious<br>-Somewhat serious<br>-Not very serious<br>-Not serious at all<br>-Don’t know
Q19	Why did you select [response question 18] when answering the previous question?		Open-ended CSP	-	Global environmental problems	-
Q20	And how serious do you consider the problem of the pollution of rivers, lakes, and oceans to be for the world as a whole?	4-point Likert	Closed-ended	WVS5	Global environmental problems	-Very serious<br>-Somewhat serious<br>-Not very serious<br>-Not serious at all<br>-Don’t know
Q21	Why did you select [response question 20] when answering the previous question?		Open-ended CSP	-	Global environmental problems	-
Q22	How interested are you in environmental issues?	5-point Likert	Closed-ended	-	-	-Very interested<br>-Somewhat interested<br>-Neither interested nor disinterested<br>-Somewhat disinterested<br>-Very disinterested
Q23	How concerned are you about environmental issues?	5-point Likert	Closed-ended	-	-	-Very concerned<br>-Somewhat concerned<br>-Neither concerned nor unconcerned<br>-Somewhat unconcerned

						-Very unconcerned
Q24	Which of the following statements do you think is most important?	4-point	Closed-ended	WVS5	-	-Maintaining order in the nation<br>-Giving people more say in important government decisions<br>-Fighting rising prices<br>-Protecting freedom of speech
Q25	And which statement is the second most important?	4-point	Closed-ended	WVS5	-	-Maintaining order in the nation<br>-Giving people more say in important government decisions<br>-Fighting rising prices<br>-Protecting freedom of speech
	Let's finish with some questions about you		Introduction socio-economic questions	-	-	-
Q26	Are you… (male, female, or other)?	3-point	Closed-ended	ISSP BVQ 2018	-	-Male,<br>-Female,<br>-Or other
Q27	How old are you?		Open-ended		-	-
Q28	In which state do you live?		Closed-ended		-	-dropdown list, 1-37 for Indian respondents, 1-53 for US respondents
Q29	Do you belong to a religion or		Closed-ended	ISSP	-	-I do not belong to a

	religious denomination? If yes, which one?		(other please specify open)	BVQ 2018		denomination<br>-Roman Catholic<br>-Protestant<br>-Orthodox (Russian/Greek/etc.)<br>-Jew<br>-Muslim<br>-Buddhist<br>-Other, please specify
Q30	What is your highest level of completed education?		Closed-ended	ISCED	-	-Less than high school<br>-Some high school, no diploma<br>-High school or equivalent (GED)<br>-Associate degree (AA, AS)<br>-Bachelor’s degree<br>-Master’s degree<br>-Professional degree (MD, DDS, JD)<br>-Doctorate degree
Q31	Compared with other American/Indian households in general, how high would you say your household income is?		Closed-ended	2021 GSS codebook	-	-Far below average<br>-Below average<br>-Average<br>-Above average<br>-Far above average
Q32	Would you describe the place where you live as…	5-point	Closed-ended	ISSP BVQ 2018	-	-A big city<br>-The suburbs or outskirts of a big city

						-A small city or town<br>-A country village<br>-A farm or home in the country
Q33	Previously in the survey, we asked you some questions regarding environmental problems in your own community. Could you describe the community you had in mind when answering these questions? (multiple answers possible)		Closed-ended (multiple options possible, other please specify)	-	-	-Your neighborhood, town, or city<br>-Your friends and family<br>-Your colleagues or fellow students<br>-Other, please specify
Q34	On which device did you complete this questionnaire?	4-point	Closed-ended		-	-PC/ laptop<br>-Tablet<br>-Smartphone<br>-Other
Q35	How would you rate the survey overall?		Closed-ended		-	-Very good<br>-Good<br>-Average<br>-Poor<br>-Very poor
Q36	Do you have any further comments about this survey?		Open-ended		-	-

WVS5: World Value Survey, wave 5; WTP: willingness to pay, SP: specific probe, CSP: category selection probe, CP: comprehension probe, ISSP: international social survey program, BVQ: background variable questionnaire, ISCED: international standard classification of education, GSS: general social survey.
Displayed choice options for religion and education as in the US questionnaire. For Indian options, see Indian questionnaires.
